# Understanding the problem of chronic kidney disease in the Republic of Kazakhstan: the study on prevalence and patients' health behaviors

**DOI:** 10.3389/fepid.2026.1685648

**Published:** 2026-06-03

**Authors:** Yerlan Burkutov, Yernur Zikiriya, Olzhas Zhandossov, Dinara Makhanbetkulova

**Affiliations:** 1 Department of Population Health and Social Sciences, Kazakhstan’s Medical University “KSPH”, Almaty, Kazakhstan; 2 Department of Nursing, Asfendiyarov Kazakh National Medical University, Almaty, Kazakhstan

**Keywords:** chronic kidney disease, health literacy, hemodialysis, Kazakhstan, prevalence, self-management

## Abstract

**Background:**

There is a lack of epidemiological data on the prevalence of chronic kidney disease (CKD) and hemodialysis, as well as associated health behaviors in Central Asia, although this information is needed for the management of healthcare services. Therefore, this study was undertaken to analyze the prevalence of CKD and hemodialysis in the population of selected provinces of Kazakhstan over the period from 2017 to 2019. In addition, we investigated health literacy and self-management of patients with CKD.

**Methods:**

This study had a two-stage design. The first stage was a retrospective cross-sectional study, which relied on the data obtained from the electronic database maintained by the Republican Center for Health Development. Point and period prevalence of CKD and hemodialysis were calculated per million population (PMP). The second stage had a prospective cross-sectional design and involved 79 CKD patients presenting at a single nephrology center.

**Results:**

The point prevalence of CKD was the highest in 2017 ranging from 137.0 PMP in North Kazakhstan province to 388.8 PMP in Turkestan province. The point prevalence of hemodialysis was higher than that of CKD and ranged from 359.3 PMP (East Kazakhstan province, 2017) to 629.3 PMP (Kostanay province, 2018). There was a growth in the point prevalence of hemodialysis over the period of 3 years in all provinces, apart from Kostanay. CKD patients had suboptimal knowledge of their disease and less than adequate self-management skills with certain gender-specific differences.

**Conclusion:**

Additional studies are required to address the knowledge gap concerning determinants of CKD in the population of Kazakhstan.

## Introduction

Chronic kidney disease (CKD) or chronic renal failure is described as impairment of kidney structure and/or function, which lasts for a period of three months or longer. The alternative definition is an estimated glomerular filtration rate not exceeding 60 mL per minute per 1.73 m^2^. The diagnosis of CKD is made on the basis of imaging studies or renal biopsy and laboratory findings that include changes in the urinary sediment or increased urinary albumin excretion rates. Being a progressive loss of kidney function secondary to any renal disorder, CKD finally leads to the need for renal replacement therapy (RRT) that can be provided via dialysis or kidney transplantation (1). CKD remains a significant public health problem, despite recent advances in diagnosis and management.

According to the “Kidney Disease: Improving Global Outcomes (KDIGO) 2012 Clinical Practice Guidelines”, CKD is classified into 6 categories on the basis of glomerular filtration rate and 3 stages depending on the levels of albuminuria. Each stage of CKD could be further categorized in accordance with the urinary albumin-creatinine ratio in an early morning urine sample (2). Because KDIGO is the improved classification system, it is good for identification of prognostic indications associated with reduced kidney function and increased albuminuria. Still, it may result in over-diagnosis of CKD, which has negative impact on many patients, in particular, the elderly.

As the nature of CKD to progress, it predisposes the affected individuals to increased mortality due to all causes. Besides, CKD is associated with decreased longevity and quality of life, as well as with broad range of complications from the side of cardiovascular system. Inevitably, this leads to additional costs and imposes an extra burden on healthcare system due to the management of secondary disorders. Such, it was estimated that CKD ranks 12th in the causes of death and there was a dramatic rise in overall CKD mortality over the past decade (3). This is why many nations have considered CKD to be the socially significant non-communicable disease and a public health priority. However, this is not a matter of fact in the countries belonging to the post-Semashko model of healthcare system, like the Republic of Kazakhstan (hereafter—Kazakhstan). As a result, there is lack of clear policies on controlling CKD, its causes and sequences, which necessitates additional research. Also, little is known about health behaviors of CKD patients. Having these data in hand, it would be possible to design and implement robust interventions targeted at CKD prevention and management, as well as to envisage the necessary RRT services. Therefore, this study was undertaken to analyze the prevalence of CKD and hemodialysis in the population of selected provinces of Kazakhstan over the period from 2017 to 2019. In addition, we investigated health literacy and self-management of patients with CKD.

## Materials and methods

### Study on the prevalence of CKD and hemodialysis

This part of our study had a retrospective cross-sectional design and relied on the data obtained from the electronic database maintained by the Republican Center for Health Development (RCHD). The nation-wide database is the country's largest data source and incorporates information on a broad spectrum of health conditions. The RCHD established a country-wide net of branches that are present in each country province in order to collect health statistics on the basis of medical codes envisaged by the International Classification of Diseases, 10th revision (ICD 10). More information on operation of this electronic database could be found elsewhere (4, 5). It should be noted that the RCHD database captures diagnosed and recorded cases within the healthcare system rather than true population-based prevalence. Therefore, the estimates derived from ICD-10 codes should be interpreted as administrative healthcare-based rates and may underrepresent individuals with early-stage or asymptomatic CKD who do not seek medical care.

From this database, we extracted the records of patients living in 5 provinces of Kazakhstan, representing 4 geographic zones: the country's north (North Kazakhstan and Kostanay provinces), east (East Kazakhstan province), west (West Kazakhstan province) and south (Turkestan province) (6). The records having N18 (CKD) and Z49.1 (hemodialysis) ICD-10 code were retrieved. Next, we specifically focused on the records of adult patients (aged 20 years and older). The data on the mid-year number of population in above listed provinces were extracted from the annual edition of the Demographic Yearbook, which is issued by the Agency of Statistics of Kazakhstan (7). The period 2017–2019 was selected as it represented the most recent interval with complete and consistent data available in the RCHD database at the time of analysis.

The following formula was used to calculate the annual administrative healthcare-based rate of CKD and hemodialysis:

$$\begin{array}{rl} \mathrm{Point}\;\mathrm{prevalence}\;= & \;\;\mathrm{number}\;\mathrm{of}\;\mathrm{people}\;\mathrm{diagnosed}\;\mathrm{with}\;\mathrm{CKD}\;\mathrm{in}\;a\;\\ & \;\mathrm{specific}\;\mathrm{province}\;\mathrm{within}\;a\;\mathrm{given}\;\mathrm{year}/\mathrm{mid}\text{-}\mathrm{year}\\ & \mathrm{population}\;\mathrm{in}\;a\;\mathrm{specific}\;\mathrm{province}\;\;\times \;1,000,000 \end{array}$$

The 3-year administrative healthcare-based rate (period prevalence) was calculated as follows:

$$\begin{array}{rl} \mathrm{Period}\;\mathrm{prevalence}\;= & \;\;\mathrm{number}\;\mathrm{of}\;\mathrm{people}\;\mathrm{diagnosed}\;\mathrm{with}\;\mathrm{CKD}\;\mathrm{in}\;a\\ & \mathrm{specific}\;\mathrm{province}\;\mathrm{during}\;2017\text{-}2019/\mathrm{mid}\text{-}\mathrm{year}\\ & \mathrm{population}\;\mathrm{in}\;a\;\mathrm{specific}\;\mathrm{province}\;\mathrm{over}\;\mathrm{the}\\ & \mathrm{same}\;\mathrm{time}\;\mathrm{period}\;\;\times \;1,000,000 \end{array}$$

### Study on health literacy and self-management of CKD

This component of the study had a prospective cross-sectional design and included patients with CKD attending a nephrology center in Semey, East Kazakhstan region. The diagnosis of CKD was established by an experienced nephrologist based on clinical evaluation and available medical records. Inclusion criteria were adult patients (aged ≥20 years) with documented ICD-10 codes N18 (CKD) and Z49.1 (hemodialysis). Patients with incomplete or inconsistent data were excluded from the analysis. A total of 143 patients with CKD were followed up at the center, of whom 86 agreed to participate and 79 returned completed questionnaires. All eligible and consenting patients during the study period were included in the prospective component.

The questionnaire was newly developed based on a review of previously published studies on health literacy and self-management in CKD patients (8, 9). The questionnaire was not formally validated, and pilot testing was not conducted prior to its use. The questionnaire included items related to health literacy and attitudes toward disease self-management. In addition, a socio-demographic section collected information on sex, age, educational level, and duration of follow-up. The socio-demographic characteristics of the study participants are presented in Table 1.

**Table 1:** Socio-demographic characteristics of patients enrolled in the study of health behaviors (*n* = 79).

Characteristic		*N*	%
Gender	Female	45	56.96
	Male	34	43.04
Age, years	20–40	12	15.19
	41–60	37	46.83
	61–75	25	31.64
	76 and older	4	5.06
	Nor specified	1	1.27
Education	Secondary	2	2.53
	Secondary vocational	36	45.57
	Higher	40	50.63
	Not specified	1	1.27
Duration of follow-up, years	<1	6	7.59
	1–3	9	11.39
	3–5	30	37.97
	5–10	27	34.18
	>10	7	8.86

The questions in health literacy section were grouped into four categories and referred to the medical diagnosis, etiology, symptoms and signs of CKD, as well as kidney functions in human body. All questions were formulated as statements and a patient could either agree or disagree with each statement. As for the attitudes towards self-management of CKD, they were mostly concerned with blood pressure (BP) control, i.e., BP measurements, the frequency of systolic BP exceeding 140 mmHg, consumption of salt and canned food, sports engagement, episodes of hospitalization due to high BP during the previous year, and presence of stroke, congestive heart failure, and cardiac attack in past history. A separate question in this section related to intake of all prescribed medications.

The retrospective component included multiple provinces to capture regional variation across different geographic areas of Kazakhstan. In contrast, the prospective component was conducted in a single specialized nephrology center due to feasibility considerations, including patient recruitment and data collection.

### Ethics statement

The study was approved by the Local Ethics Committee of Kazakhstan's Medical University “KSPH” (Protocol No. 1, dated June 17, 2025). Informed consent was obtained from all participants prior to inclusion in the study. All data were anonymized to ensure confidentiality.

### Statistical analysis

Annual and 3-year administrative healthcare-based rates per million population were calculated with the help of Microsoft Excel spreadsheet software. Data from the study on health literacy and self-management were analyzed with the help of IBM SPSS Statistics 23 software. As the initial stage of data analysis, the mode of data distribution was evaluated by means of the Shapiro–Wilk test. Since it was different from normal, the Pearson's chi-square test was used to test for the difference between study groups (males vs. females). The Yates chi-square test was used when the expected frequency of one or more cells in contingency table was less than 5. Significance level of *α* < 0.05 was used.

## Results

Table 2 presents characteristics of patients hospitalized for CKD during three consecutive years (2017–2019). A high proportion of emergency hospitalizations was observed across several regions and years. There was male predominance in almost all provinces with exception of 2017, when female predominance was noted in East Kazakhstan, Kostanay and North Kazakhstan provinces. Most patients were in the age group of 50–59 years, and the second commonest age group was 60–69 years. Nevertheless, there were certain provincial variations in the age structure of CKD patients. Such, the majority of patients hospitalized in West Kazakhstan province in 2019 aged 70 years and older (22.9%), followed by the age group of 40–49 years (21.7%). Interestingly, there was a substantial variation in the rate of emergency hospitalization, which exceeded 50% during nearly the entire study period. Still, the rate of emergency hospitalization was low in 3 out of 5 provinces in 2018 and 2019.

**Table 2:** Characteristics of patients from selected provinces of Kazakhstan hospitalized for CKD, 2017–2019.

Characteristics			East Kazakhstan	Kostanay	North Kazakhstan	West Kazakhstan	Turkestan
Proportion of male population (%)		2017	49.2	49.3	42.6	55.9	56.1
		2018	53.2	51.4	54.1	53.0	58.2
		2019	51.5	55.3	62.7	47.1	55.8
Proportion of different age groups (%)	20–29 years	2017	13.0	2.1	6.6	4.9	11.4
		2018	4.1	20.8	13.1	10.6	11.0
		2019	5.9	7.5	5.5	4.5	8.8
	30–39 years	2017	17.4	10.0	6.6	7.7	16.6
		2018	16.9	24.3	18.9	25.0	35.2
		2019	15.1	9.3	10.0	10.2	14.0
	40–49 years	2017	18.1	15.7	11.5	19.6	15.2
		2018	31.9	23.7	27.0	26.3	34.1
		2019	15.7	9.9	15.5	21.7	17.5
	50–59 years	2017	26.4	25.7	21.3	30.1	22.0
		2018	19.7	12.7	25.4	14.2	9.9
		2019	31.1	21.7	29.1	22.3	24.9
	60–69 years	2017	21.4	22.1	37.7	26.6	22.5
		2018	15.9	13.3	11.5	12.5	6.6
		2019	23.7	31.1	22.7	18.5	24.7
	70 years and older	2017	3.7	24.3	16.4	11.2	12.3
		2018	11.5	5.2	4.1	11.5	3.3
		2019	8.6	20.5	17.3	22.9	10.1
Proportion of emergency hospitalization (%)		2017	85.3	82.9	95.1	60.6	77.5
		2018	15.6	15.6	69.7	75.4	4.4
		2019	19.8	8.7	3.6	70.8	75.7

**Figure 1:**
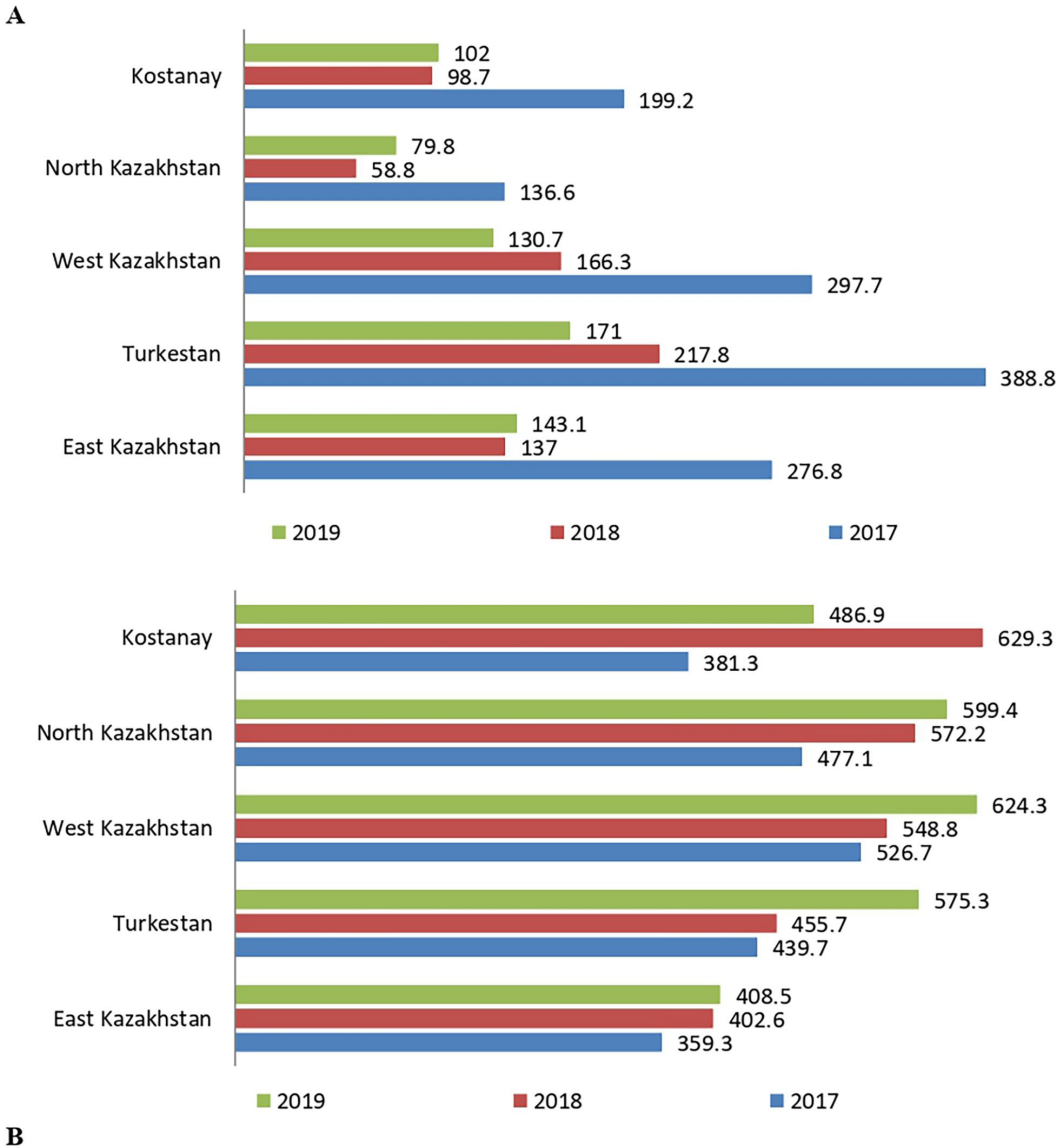
Point prevalence of chronic kidney disease **(A)** and haemodialysis **(B)** in the population of selected provinces of Kazakhstan, 2017–2019 (per million population aged ≥20 years).

**Figure 2:**
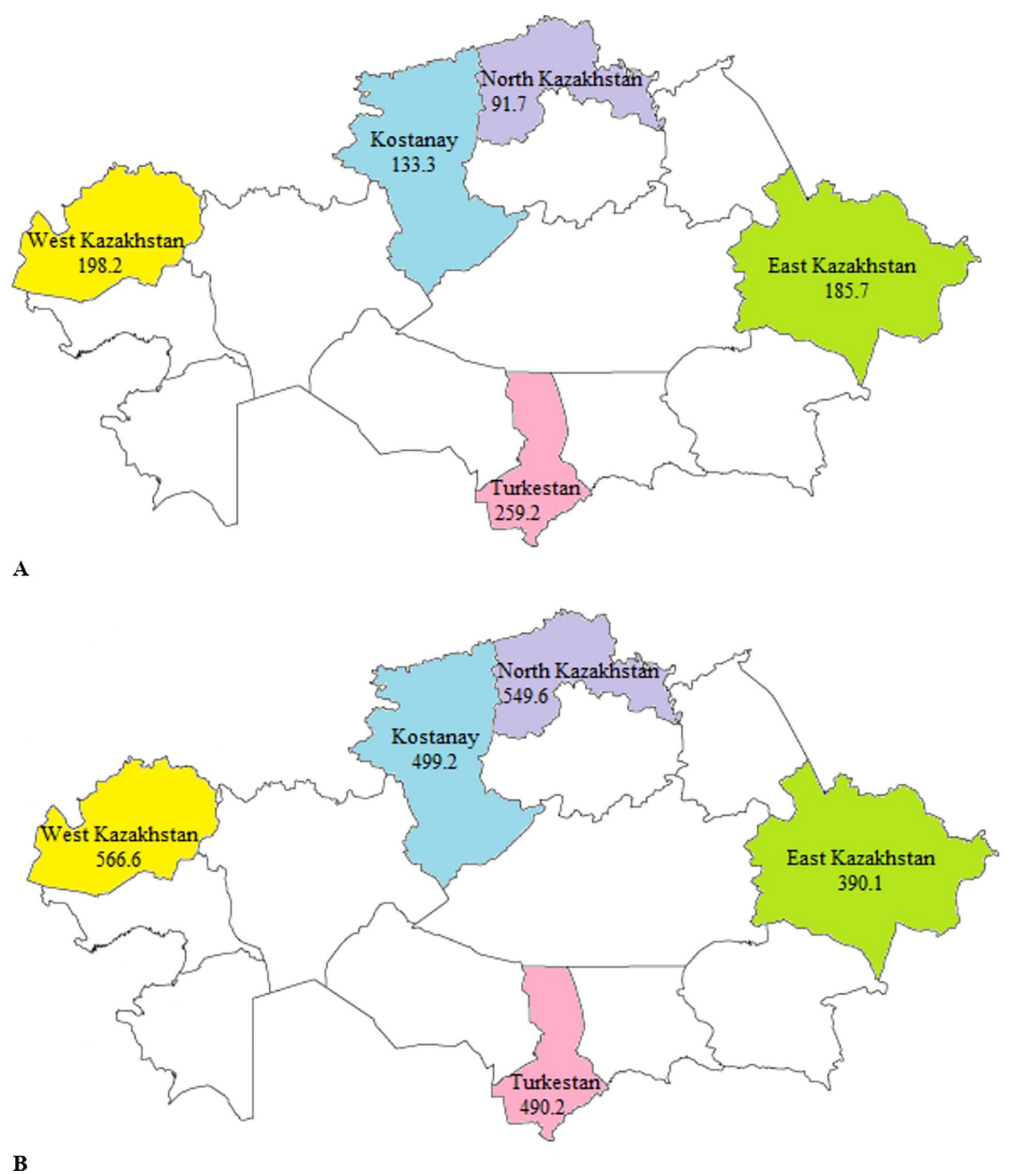
Period prevalence (2017–2019) of chronic kidney disease **(A)** and haemodialysis **(B)** in the population of selected provinces of Kazakhstan (per million population aged ≥20 years).

The point prevalence of CKD was the highest in 2017 ranging from 137.0 PMP in North Kazakhstan province to 388.8 PMP in Turkestan province. The lowest point prevalence of CKD was seen in North Kazakhstan province in 2018 and equaled 58.8 PMP. However, the point prevalence of hemodialysis was higher than that of CKD and ranged from 359.3 PMP (East Kazakhstan province, 2017) to 629.3 PMP (Kostanay province, 2018). There was a growth in point prevalence of hemodialysis over the period of 3 years in all provinces, apart from Kostanay, where the highest rate was seen in 2018 (Figure 1). As for the period prevalence of CKD, the lowest rate was observed in North Kazakhstan province (91.7 PMP) and the highest rate was seen in Turkestan province (259.2 PMP). Meanwhile, the period prevalence of hemodialysis showed the highest rate in West Kazakhstan province (566.6 PMP) and the lowest rate was observed in East Kazakhstan province (390.1 PMP) (Figure 2). It should be noted that the lower recorded rates of CKD compared to hemodialysis likely reflect the hospital-based nature of the data and potential underdiagnosis of early-stage CKD rather than true population-level patterns.

Table 3 presents information on health literacy of CKD patients. There was a significant difference between male and female patients in the knowledge of tests used for CKD diagnosis as males were better informed on the role of plasma creatinine. However, female patients were significantly more aware of kidney functions in human body. Significantly more male patients measured their BP, which might be attributed to the fact that a higher proportion of them had systolic BP exceeding 140 mmHg most of the time. Still, male patient consumed salt with food and canned food more often than female patients did. Such complications of arterial hypertension as stroke, congestive heart failure, and cardiac attack in past history were more commonly observed in male patients with CKD. Nevertheless, none of the above listed differences reached the level of statistical significance (Table 4).

**Table 3:** Health literacy of patients with chronic kidney disease (*n* = 79).

Categories		Male		Female		Test of difference	
		*N*	%	*N*	%	*χ* ^2^	*P*-value
Medical diagnosis	Plasma creatinine	20	58.82	6	13.33	18.151	<0.01
	Albuminuria	12	35.29	19	42.22	0.390	0.53
	Other tests	1	2.94	20	44.44	15.033	<0.01
Etiology	Drug- and toxin-induced nephropathy	5	14.71	1	2.22	2.706	0.1
	Kidney stones	0	0.00	2	4.44	0.272	0.60
	Glomerulonephritis	3	8.82	12	26.67	2.933	0.09
	Arterial hypertension	11	32.35	16	35.56	0.088	0.77
	Diabetes mellitus	9	26.47	8	17.78	0.867	0.35
Kidney functions	Blood pressure control	5	14.71	18	40.0	6.004	0.01
	Blood filtration	7	20.59	20	44.44	4.899	0.03
	Maintenance of bone health	9	26.47	24	53.33	5.746	0.01
Symptoms and signs	Fluid overload	13	38.23	9	20.00	3.205	0.07
	Fever	9	26.47	12	26.67	0.0004	0.98
	Nausea/vomiting	13	38.23	10	22.22	2.406	0.12
	Anorexia	10	29.41	13	28.89	0.003	0.96
	Weakness	10	29.41	12	26.67	0.073	0.79

**Table 4:** Self-management of patients with chronic kidney disease (*n* = 79).

Categories		Male		Female		Test of difference	
		*N*	%	*N*	%	*χ* ^2^	*P*-value
Blood pressure measurements	Yes	24	70.59	17	37.78	8.352	<0.01
	No	10	29.41	28	62.22		
Systolic blood pressure exceeding 140 mmHg	Most of the time	8	23.53	10	22.22	2.347	0.31
	Sometimes	14	41.18	12	26.67		
	Unaware	12	35.29	23	51.11		
Consumption of salt with food	Every food intake	7	20.59	6	13.33	4.164	0.24
	Frequently	9	26.47	9	20.00		
	Sometimes	7	20.59	19	42.22		
	Never	11	32.35	11	24.44		
Consumption of canned food	Every day	5	14.70	2	4.44	1.467	0.69
	Few times a week	7	20.59	8	17.78		
	Sometimes	9	26.47	15	33.33		
	Never	13	38.23	20	44.44		
Sports engagement	Daily	6	17.65	7	15.56	2.610	0.62
	Three times a week	11	32.35	20	44.44		
	Once a week	9	26.47	12	26.67		
	Once a month	8	23.53	4	8.89		
	Never	0	0.00	2	4.44		
Hospitalization due to high blood pressure	Yes	22	64.71	29	64.44	0.071	0.96
	No	10	29.41	14	31.11		
	No reply	2	5.88	2	4.44		
Stroke in past history	Yes	3	8.82	2	4.44	0.106	0.74
	No	31	91.18	43	95.56		
Congestive heart failure in past history	Yes	19	55.88	19	42.22	1.448	0.23
	No	15	44.12	26	57.78		
Cardiac attack in past history	Yes	12	35.29	13	28.89	0.367	0.54
	No	22	64.71	32	71.11		
Intake of prescribed medication	Yes	31	91.18	42	93.33	0.005	0.94
	No	3	8.82	3	6.67		

## Discussion

This epidemiological study had a two-stage design. The first stage was undertaken to describe administrative healthcare-based rates of CKD and hemodialysis in population of the selected provinces of Kazakhstan, representing geographic north, south, east and west, while the second stage was carried-out to investigate health behaviors in CKD patients. In general, the period prevalence of CKD was characterized by mild variability with a significant time variation in the point prevalence during 3 years of observation. Both CKD and hemodialysis patients were rather young (the predominant age group affected was 50–59 years). Meanwhile, there were relatively few patients in the age category of 70 years and older, which indicates suboptimal longevity of these patients. As for the health behaviors, CKD patients had suboptimal knowledge of their disease and less than adequate self-management skills with certain gender-specific differences. An important methodological observation in this study is that the recorded CKD rates were lower than the rates of hemodialysis, which is not expected in a population-based setting. This discrepancy is most likely explained by the hospital-based nature of the data and the structure of the administrative registry. Patients undergoing hemodialysis are systematically captured due to the need for regular treatment, whereas individuals with earlier stages of CKD are less likely to be diagnosed, hospitalized, or recorded in the database. In addition, changes in hospitalization criteria and healthcare system organization may have contributed to reduced recording of non-dialysis CKD cases. Therefore, this finding should be interpreted as an artifact of data collection and healthcare utilization rather than a reflection of the true epidemiological relationship between CKD and end-stage renal disease. The single-center design of the prospective component should also be considered when interpreting the findings, as it may limit their generalizability to other regions. Based on the estimates of the World Health Organization (WHO), the average life expectancy in Kazakhstan is not high and equals 59 years for men and 70 years for women and this could be attributed to a variety of factors, including poor control over such non-communicable chronic diseases (NCDs) as cancer, cardiovascular and respiratory disease, and diabetes (10). Arterial hypertension and diabetes mellitus are the known risk factors for CKD (8) and their prevalence in the general public constitutes 27% and 12%, respectively (11). In Kazakhstan, NCDs account for 86% of all deaths and incidence of CKD grows rapidly (10).

In general, life expectancy of CKD patients depends on the estimated glomerular filtration ration (eGFR) and Turin with co-authors constructed life expectancy tables for male and female patients with CKD on the basis of a large population-based registry. These tables set the following thresholds for eGFR: ≥60, 45–59, 30–44 and 15–29 mL per minute per 1.73 m^2^ and there is no surprise that life expectancy declines with each age group of worse renal function. For instance, as compared with the individuals having eGFR: ≥60 mL/min/1.73 m^2^, life expectancy is reduced by nearly 65% in those individuals who present with an eGFR of 15–29 mL/min/1.73 m^2^ (12). In addition, the absence of key clinical parameters such as estimated glomerular filtration rate, CKD stage, and albuminuria limits interpretation of disease severity and reduces comparability with studies using standardized CKD classification frameworks.

CKD is strongly associated with increased prevalence of cardiovascular disease that is recognized as the major risk factor for CKD-related mortality. In Canada, an increase in the death rate from CKD was associated with the presence of cardiovascular comorbidity, in particular, ischemic heart disease. A decrease in eGFR influences the increased cardiovascular mortality, which reaches the level of 44% when eGFR drops to 15–29 mL per minute per 1.73 m^2^. Infectious disease is the second strongest predictor for CKD-related mortality (13). Apart from eGFR, high albumin/creatinine ratio (ACR) exceeding 10 mg/g is another independent predictor for cardiovascular mortality in the general public (14).

Traditionally, patients with end-stage renal disease (ESRD) are eligible for RRT and hemodialysis is the commonest treatment option available in Kazakhstan. All services are provided to patients for free and National Health Insurance Foundation is the only healthcare financing agency. Since the early 2010s, the country's government was committed to the establishment of public-private partnership in healthcare and this helped to improve availability of and access to hemodialysis services for patients living in different geographic regions. However, most services operate in urban areas and rural hemodialysis centers have only recently become available. As a result, there is a backlog of ESRD patients living in rural areas and this public health issue has not been met yet (15).

Our findings suggest a decreasing trend in recorded CKD rates and an increasing trend in recorded hemodialysis rates within the healthcare system. These findings should be interpreted with caution because the study relied on administrative hospital- and registry-based data rather than population-based screening data. The use of ICD-10 codes introduces potential selection and ascertainment bias, since only individuals who accessed healthcare services and received a documented diagnosis were captured in the database. As a result, patients with early-stage or asymptomatic CKD who did not seek medical care were likely underrepresented. Therefore, the observed CKD rates may underestimate the true burden of non-dialysis CKD in the community, whereas the increasing hemodialysis rates may more directly reflect the expansion and utilization of renal replacement therapy services in Kazakhstan. In addition, the high proportion of emergency hospitalizations observed across several regions and years may reflect characteristics of healthcare system organization and access to care in Kazakhstan. Limited access to early diagnosis and outpatient management may result in delayed presentation of patients, leading to a higher reliance on emergency hospital services. Furthermore, administrative and admission practices may influence the classification of hospitalizations as emergency cases.

Kazakhstan is not unique in the growing trend of RRT services as many countries experienced a stable increase over the past decade. The annual growth rate constituted 9.99% in Russia, 12.51% in United Kingdom, 11.87% in Spain, 13.37% in Poland, 13.88% in Italy, and 9.65% in Turkey. Besides, all of the above referenced countries, except for Russia, had higher prevalence of RRT services PMP, as of 2018. For example, this rate equaled 972 in United Kingdom, 1,284 in Spain, 788 in Poland, 1,137 in Italy, and 957 in Turkey. Such countries as Canada and the USA had even higher prevalence of RRT services PMP: 1,405.3 and 2,203.5, respectively. Interestingly, both Canada and the USA had the most modest upward trend in RRT services PMP: 2.93% and 3.0%, respectively (16). Perhaps, one might be tempted to conclude that these countries already reached the upper limit of service provision and hence fully meet the demands. Recent evidence from post-Soviet countries also highlights persistent public health challenges, including the growing burden of non-communicable diseases and the need to strengthen healthcare systems to ensure equitable access to services such as renal replacement therapy. These findings further support the observed expansion and variability of RRT services across different settings (17, 19).

A range of international studies have explored health literacy among patients with CKD and its association with self-management behaviors. Taylor et al. conducted a systematic review examining the relationship between health literacy and patient outcomes, demonstrating that low health literacy is associated with increased rates of hospital admissions, cardiovascular events, mortality, and poor adherence to hemodialysis. However, the evidence for a causal relationship remains limited, as most studies were of moderate methodological quality. Consistent with our findings, patients with low health literacy have a reduced ability to interpret test results, which may lead to poorer control of cardiovascular risk factors, lower treatment adherence, and greater reliance on urgent and emergency care services (18). However, these findings should be interpreted with caution due to the relatively small sample size, single-center design of the prospective component, and the absence of adjustment for multiple statistical comparisons, which may increase the risk of type I error.

This study has several limitations. First, the retrospective component relied on hospital- and registry-based administrative data rather than population-based screening data. Therefore, the identified CKD cases reflect diagnosed and recorded cases within the healthcare system and should not be interpreted as true population prevalence. The use of ICD-10 codes may have introduced selection and ascertainment bias, particularly through underrepresentation of individuals with early-stage or asymptomatic CKD who did not seek medical care. Second, key clinical variables required for proper CKD classification, including estimated glomerular filtration rate (eGFR), CKD stage, and albuminuria, were not available in the dataset. This precluded stratification by disease severity and limits clinical interpretation, as well as comparability with existing literature. Third, detailed information on comorbidities, lifestyle risk factors, and socioeconomic characteristics was not available, which limited the ability to perform stratified analyses. Fourth, the prospective component was conducted on a relatively small sample (*n* = 79) from a single center, which limits the generalizability of findings related to health literacy and self-management. A formal sample size calculation was not performed for the prospective component, as all eligible and consenting patients during the study period were included. In addition, multiple statistical comparisons were performed in the analysis of health literacy and self-management variables without formal adjustment for multiple testing, which may increase the risk of type I error. Nevertheless, the retrospective component relied on a large regional dataset, and the selected nephrology center represents a typical service for this patient group. Future studies based on longer observation periods and larger populations would provide more robust estimates of CKD burden and trends.

## Conclusion

Nowadays, there is lack of systematic surveillance on prevalence and causes of CKD in Central Asian countries, as well as associated health behaviours, while these data are essential for planning of robust interventions targeted at kidney disease management. Availability of such information may lead to fair distribution of healthcare resources, in particular, RRT services. Obviously, establishment of a national registry on CKD could help to overcome this knowledge gap. Besides, certain patient-targeted interventions have to be envisaged to improve their knowledge of the underlying condition and self-management skills as better health behaviours are associated with less complications, decreased healthcare use and mortality.

## Data Availability

Publicly available datasets were analyzed in this study. This data can be found here: our data in excel.
